# The AdcACB/AdcAII system is essential for zinc homeostasis and an important contributor of *Enterococcus faecalis* virulence

**DOI:** 10.1080/21505594.2022.2056965

**Published:** 2022-03-28

**Authors:** Ling Ning Lam, Debra N. Brunson, Jonathan J. Molina, Ana L. Flores-Mireles, José A. Lemos

**Affiliations:** a Department of Oral Biology, University of Florida College of Dentistry, Gainesville, FL, USA; b Department of Biological Sciences, University of Norte Dame, Notre Dame, IN, USA

**Keywords:** *Enterococcus faecalis*, zinc uptake, nutritional immunity, virulence, pathogenesis

## Abstract

Bacterial pathogens require a variety of micronutrients for growth, including trace metals such as iron, manganese, and zinc (Zn). Despite their relative abundance in host environments, access to these metals is severely restricted during infection due to host-mediated defense mechanisms collectively known as nutritional immunity. Despite a growing appreciation of the importance of Zn in host-pathogen interactions, the mechanisms of Zn homeostasis and the significance of Zn to the pathophysiology of *E. faecalis*, a major pathogen of nosocomial and community-associated infections, have not been thoroughly investigated. Here, we show that *E. faecalis* encoded ABC-type transporter AdcACB and an orphan substrate-binding lipoprotein AdcAII that work cooperatively to maintain Zn homeostasis. Simultaneous inactivation of *adcA* and *adcAII* or the entire *adcACB* operon led to a significant reduction in intracellular Zn under Zn-restricted conditions and heightened sensitivity to Zn-chelating agents including human calprotectin, aberrant cell morphology, and impaired fitness in serum *ex vivo*. Additionally, inactivation of *adcACB* and *adcAII* significantly reduced bacterial tolerance toward cell envelope-targeting antibiotics. Finally, we showed that the AdcACB/AdcAII system contributes to *E. faecalis* virulence in a *Galleria mellonella* invertebrate infection model and in two catheter-associated mouse infection models that recapitulate many of the host conditions associated with enterococcal human infections. Collectively, this report reveals that high-affinity Zn import is important for the pathogenesis of *E. faecalis* establishing the surface-associated AdcA and AdcAII lipoproteins as potential therapeutic targets.

## Introduction

Iron (Fe), manganese (Mn), and zinc (Zn) are essential trace metals to all forms of life. They serve structural, catalytic, and regulatory functions to metalloproteins involved in a variety of biological processes [1–4]. As a result of this essentiality, hosts deploy a variety of strategies to deprive access of invading pathogens to trace metals, an active process termed nutritional immunity [2,5–8]. To date, the best characterized nutritional immunity strategy is based on mobilization of metal-chelating proteins to the infection site by host immune cells [2]. Among them, calprotectin, a member of the S100 protein family produced by neutrophils and other types of immune cells that is secreted in large quantities during infection and inflammatory processes, is the main host protein responsible for Mn^2+^ and Zn^2+^ sequestration [9–11]. To overcome trace metal limitation, microbial pathogens evolved effective metal-scavenging systems that include expression of surface-associated high-affinity metal uptake systems and, in some bacterial species, synthesis and trafficking of organic extracellular molecules known as metallophores [8].

Although the importance of Fe in host-pathogen interactions has been extensively examined [6], the role of Mn and Zn in host-pathogen interactions and the mechanisms utilized by bacteria to maintain their cellular levels and ratios properly balanced are less understood [7,12–17]. The second most abundant trace metal in vertebrates, Zn, is estimated to be incorporated into approximately 5% of the bacterial proteome and plays structural and catalytic roles in multiple biological processes [18,19]. In bacteria, Zn acquisition under severe Zn-restricted conditions such as those that can be encountered in host environments depends on the activity of surface-associated Zn uptake systems from the ATP-binding cassette (ABC) transporter family (reviewed in Refs. 20–22). Moreover, major human pathogens such as *Pseudomonas aeruginosa* and *Staphylococcus aureus* produce Zn-binding metallophores, also known as zincophores [23].

To date, the contributions of Zn uptake systems to virulence have been demonstrated in a number of bacterial species, including several Gram-positive pathogens that are phylogenetically related to *Enterococcus faecalis*, the subject organism of the present study. In *S. aureus*, inactivation of either the ABC-type transporter AdcABC, the staphylopine (Stp) zincophore, or its cognate multi-metal transporter CntABCDF was sufficient to impair bacterial growth under Zn-restricted conditions *in vitro* [24]. Loss of both AdcABC and Stp/CntABCDF systems resulted in further growth impairment under Zn-restricted conditions and attenuated virulence in a mouse retro-orbital infection model [24]. In streptococci, which to date reportedly do not synthesize zincophores, Zn acquisition is mediated by the ABC-type transporter AdcABC. In addition to AdcABC, streptococcal species encode an additional *adcA* homologue, known as *adcAII* (reviewed in [25]), coding for a second Zn-binding lipoprotein. In *Streptococcus pyogenes*, strains lacking *adcC*, *adcA*, or *adcAII* grew poorly in the presence of purified human calprotectin and displayed attenuated virulence in a necrotizing fasciitis mouse model [26] and in a humanized-plasminogen skin infection mouse model [27]. Moreover, *S. pyogenes* Δ*adc* strains retained wild-type strain levels of virulence in calprotectin-negative (*S100a9*^−/−^) mice [26], which validates the central role of calprotectin in host-mediated Zn sequestration and protection against bacterial infection. Similarly, virulence of *Streptococcus pneumoniae* Δ*adcA*Δ*adcAII* and *Streptococcus agalactiae* Δ*adcA*Δ*adcAII*Δ*lmb* (*lmb* encodes for a 3^rd^ Zn-binding lipoprotein) strains was significantly attenuated in mouse models of systemic and nasopharyngeal colonization [16,28]. Finally, in the oral pathogen *S. mutans*, one of the few streptococci that do not encode the orphan *adcAII* gene, inactivation of the *adcABC* system significantly impaired bacterial growth under Zn-restricted conditions and reduced bacterial colonization of the dental biofilm in a rat model [12,29].

A commensal of the gastrointestinal (GI) tract, *E. faecalis*, is also a prevalent opportunistic pathogen of localized and systemic infections, including but not limited to infective endocarditis, catheter-associated urinary tract infections (CAUTI), and wound infections [30–33]. A major virulence trait of *E. faecalis* is its remarkable capacity to adapt to adverse conditions in the GI tract (their natural host environment) and several other host tissues, and to survive exposure to hospital-grade disinfectants and antibiotic treatments [34,35]. Because very little is known about the mechanisms of Zn homeostasis in enterococci, we sought to characterize the Zn acquisition systems of *E. faecalis* in this study. Similar to streptococci, the core genome of *E. faecalis* encodes for a conserved AdcACB system (originally annotated as *znuACB*) and an orphan substrate-binding lipoprotein AdcA-II that is annotated as *adcA*. In this report, we isolated a panel of *E. faecalis* ∆*adc* strains, including strains lacking every *adc* gene (∆*adcACB∆adcAII*) or both genes coding for the substrate-binding lipoproteins (∆*adcA∆adcAII*), and then used these mutants to define the role of AdcACB and AdcAII in *E. faecalis* pathophysiology. Our results revealed that simultaneous inactivation of *adcA* and *adcAII* or of the entire *adcACB* operon yielded the most impactful phenotypes, which included severe growth/survival defects in the presence of calprotectin or in human serum, and attenuated virulence in both invertebrate and vertebrate infection models. We also discovered that the inability to maintain Zn homeostasis diminished the recognized high tolerance of *E. faecalis* to antibiotics that target the cell envelope. Collectively, this study reveals that AdcACB and AdcAII work cooperatively to maintain *E. faecalis* Zn homeostasis during infection such that the surface-associated AdcA and AdcAII lipoproteins can be considered potential targets for the development of antimicrobial interventions.

## Results

### AdcACB and AdcAII work in concert to promote growth under Z*n*-restricted conditions

**Figure 1.**
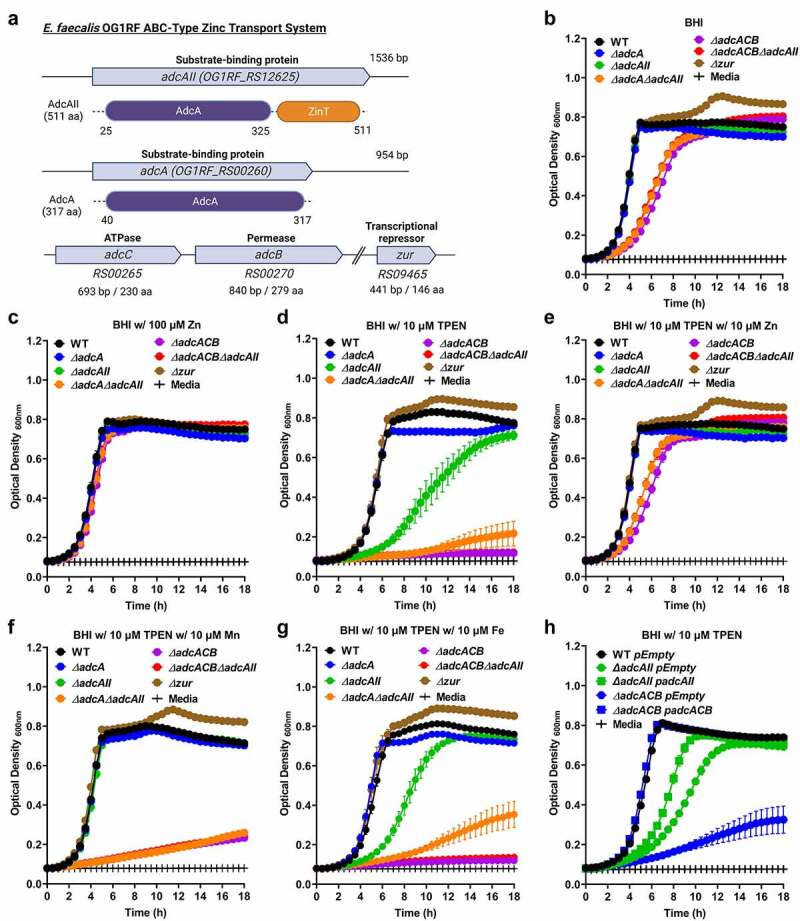
Growth characteristics of *E. faecalis* and its Z*n*-deficient mutants under Z*n*-restricted conditions. (a) Schematic of the gene locus of the Zn transport system in *E. faecalis* OG1RF core genome. Growth curves of *E. faecalis* wild type (WT) and its isogenic mutants in BHI (b), BHI supplemented with 100 µmZnSo_4_ (c), 10 µm TPEN (d), combination of TPEN and ZnSo_4_ (e), TPEN and MnSo_4_ (f) or TPEN and Fe SO_4_(g). In (b) and (d-f), data points represent the average of nine biological replicates. Finally, the growth curve of *E. faecalis* wild type (WT) and genetically complemented ∆*adc* mutants in BHI supplemented with 10 µm TPEN (h). In (c) and (g), data points represent the average of six biological replicates. Error bar represents the standard error of margin (SEM). Statistical analysis was performed using simple linear regression ofthe exponential growth phase, and slope of each mutant’s growth kinetics was compared with that of the parent strain.

Using the NCBI BLASTn tool, we identified the genes coding for the highly conserved ABC-type transporter AdcACB (*OG1RF_RS00260-RS00270*), the orphan substrate-binding AdcAII lipoprotein (*OG1RF_RS12625*) and the transcriptional repressor Zur (*OG1RF_RS09465*) in the *Enterococcus faecalis* OG1RF genome (GenBank: CP002621.1) (Figure 1a). The translated gene products of *OG1RF_RS00260* (AdcA; Accession ID: AEA92738.1, protein ID: WP_002367576.1) and *OG1RF_RS12625* (AdcAII; Accession ID: AEA95159.1, protein ID: WP_002392710.1) display respectively, 57% and 64% amino acid similarity to the *S. pneumoniae* AdcA and 42% and 39% similarity to *S. pneumoniae* AdcAII [16] (**Figure S1**). Pairwise alignment between *E. faecalis* AdcA and AdcAII also revealed 53% similarity, indicative of functional redundancy (**Figure S1)**. AdcAII, the larger of the two Zn-binding lipoproteins of *E. faecalis*, contains a ZinT-like domain at the C-terminus that is also observed in the *S. pneumonia* AdcA and was shown to mediate Zn binding via the so-called trap door mechanism [17,36–38]. By contrast, the *S. pyogenes* AdcA utilizes two domains for Zn binding although it is structurally more distinct from *E. faecalis* OG1RF AdcA/AdcAII with 36% and 34% similarity, respectively (**Figure S1**) [39]. Using AlphaFold and Chimera to predict protein structures, we found that *S. pneumoniae* R6 AdcA (NP_359566.1) and AdcAII (NP_358500.1) structurally overlap with *E. faecalis* AdcAII (WP_002392710.1) and AdcA (WP_002367576.1), respectively (**Figure S2**). Moreover, the hinge region identified for Zn binding in *S. pneumoniae* AdcA [38] and *S. pyogenes* AdcA [39] was present in both *E. faecalis* AdcA and AdcAII (**Figure S2**). In previous transcriptome-based studies conducted with *E. faecalis* strain V583, the *adcABC* (originally annotated as *znuABC*) and *adcAII* genes were shown to be repressed after exposure to high Zn levels and strongly induced after treatment with the Zn-chelating agent TPEN (N,N,N′,N′-tetrakis(2-pyridinylmethyl)-1,2-ethanediamine) [40,41]. Based on the presence of conserved domains and amino acid similarities with homologous systems of closely related streptococci, we renamed the *OG1RF_RS00260-OG1RF_RS00270* gene cluster *adcACB* keeping the *adcAII* designation for the lone *OG1RF_RS12625*. Of note, none of the enterococcal genomes surveyed, including *E. faecalis* OG1RF, encode biosynthetic gene clusters and cognate transporters of opine-like zincophore systems that are found in a small number of bacterial pathogens [23].

To probe the role of AdcACB and AdcAII in Zn acquisition and, most importantly, determine the significance of these systems in *E. faecalis* pathophysiology, we used a markerless in-frame deletion strategy [42] to generate strains lacking one or both substrate-binding lipoproteins (*∆adcA, ∆adcAII*, and *∆adcA∆adcAII*) and the entire *adcACB* operon alone or in combination with *adcAII* (∆*adcACB* and ∆*adcACB∆adcAII*). In addition, we isolated a strain lacking the transcriptional repressor Zur (∆*zur*). Next, we compared the ability of *E. faecalis* OG1RF (wild-type strain) and mutant derivatives to grow in BHI, a complex media that contains ~10 µM Zn [43], or in BHI supplemented with TPEN [44]. In BHI, inactivation of either *adcA* or *adcAII* alone did not impact growth kinetics or growth rates, whereas simultaneous inactivation of *adcA* and *adcAII* (*∆adcA∆adcAII*) or the entire *adcACB* operon alone (∆*adcACB*) or in combination with *adcAII* (∆*adcACB∆adcAII*) resulted in slower growth rates without affecting final growth yields (Figure 1b). Inactivation of the *zur* regulator did not affect growth rates but led to a slight increase in the final growth yield in BHI (Figure 1b). BHI supplementation with 100 µM ZnSO_4_ (10-fold in excess of labile Zn pools in BHI media) restored the growth defects of ∆*adcA∆adcAII*, ∆*adcACB*, and ∆*adcACB∆adcAII* strains (Figure 1c). Addition of 10 µM TPEN to BHI (BHI+TPEN) minimally impacted the growth of the parent OG1RF strain (OG1RF growth is severely impaired at TPEN concentrations ≥20 µM, **Figure S3**). On the other hand, the *∆adcAII* single mutant grew poorly in BHI+TPEN, while growth of the ∆*adcACB* and ∆*adcACB∆adcAII* strains was completely inhibited by 10 µM TPEN (Figure 1d). Finally, the addition of 10 µM ZnSO_4_ to the BHI+TPEN media restored growth of the *∆adcAII*, ∆*adcACB*, and ∆*adcACB∆adcAII* strains (Figure 1e). To verify if the inhibitory effect of TPEN on growth of ∆*adc* strains was indeed Zn-specific, we tested if addition of Mn (10 µM MnSO_4_) or Fe (10 µM FeSO_4_) could also restore cell growth. With the exception of *∆adcAII* that was able to grow in BHI+TPEN after Mn supplementation, Fe or Mn supplementation did not restore growth of the other mutants in BHI+TPEN (Figure 1f-g, compared to Figure 1d). *In trans* complementation of ∆*adcACB* and of *∆adcAII* fully or partially rescued their growth defects in BHI+TPEN (Figure 1h). The reasons for Mn rescuing growth of *∆adcAII* in BHI+ TPEN and the partial complementation of *∆adcAII* are at present unknown.

**Figure 2.**
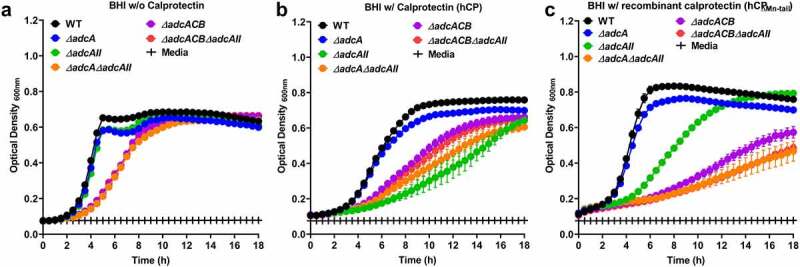
Growth characteristics of *E. faecalis* and its Z*n*-deficient mutants in the presence of calprotectin. Growth curves of *E. faecalis* OG1RF WT and its isogenic mutants in BHI supplemented with CP buffer media (a) and 150 µg ml ^−1^ of WT hCP (b) or hCp_∆mn-tail_ (c). Data points represent the average, and error bar represents the standard error of margin (SEM) of at least six biological replicates. Statistical analysis was performed using simple linear regression of exponential growth phase, and the slope of each mutant’s growth kinetics was compared with that of the parent strain.

Next, we sought to determine the ability of our panel of ∆*adc* strains to grow in the presence of human calprotectin, a potent Mn and Zn chelator. We carried out growth kinetic assays to compare the ability of wild-type (WT) and ∆*adc* mutants to grow in BHI supplemented with purified human calprotectin (hCP) or recombinant calprotectin (hCP∆_Mn-tail_) defective in Mn sequestration [10]. In the presence of hCP, growth of *∆adcA* was not significantly different when compared to WT, whereas ∆*adcAII*, ∆*adcA*∆*adcAII*, ∆*adcACB*, and ∆*adcACB*∆*adcAII* mutants displayed reduced growth or were fully inhibited by the native version of calprotectin (Figure 2a-b). While inactivation of the Mn-binding residue in hCP_∆Mn-tail_ improved growth of WT, *∆adcA* and *∆adcAII* strains, the other mutants remained highly sensitive to hCP_∆Mn-tail_ (Figure 2c). *In trans* complementation fully rescued the growth defects of ∆*adcACB* mutant in the presence of both versions of calprotectin, whereas growth of the complemented ∆*adcAII* was partially rescued (**Figure S4)**. The reason for the partial complementation of ∆*adcAII* in the presence of hCP is at present unknown. Taken together, these findings reveal that AdcABC and AdcAII work independently but cooperatively to mediate *E. faecalis* growth under Zn-restricted conditions. Based on the identical phenotypes of ∆*adcACB* and *∆adcA∆adcAII* strains, these results also indicate that both AdcA and AdcAII associate with AdcB (inner membrane permease) and AdcC (cytoplasmic ATPase) to form tripartite Zn transporters. Finally, growth kinetics in the presence of the Zn-chelating agents TPEN and calprotectin hint that AdcAII might be a more effective Zn scavenger than AdcA, at least under the more severe Zn-restricted conditions.

**Figure 3.**
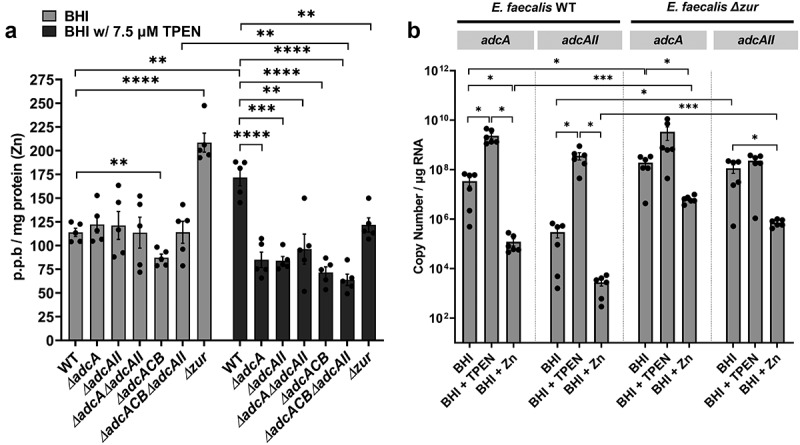
Intracellular Zn quantification and transcriptional profiles of *E. faecalis* and its Z*n*-deficient mutants. (a) ICP-OES quantifications of intracellular Zn of mid-log grown *E. faecalis* OG1RF WT and derivatives grown in BHI and BHI supplemented with 7.5 µm TPEN. Data points represent five biological replicates. Statistical analysis was performed using two-way ANOVA with Dunnett’s multiple comparison test. ** *p* ≤0.01, *** *p* ≤0.001, and **** *p* ≤0.0001. (b) Comparison of reversed transcribed cDNA copy of *adcA* and *adcAII* in *E. faecalis* OG1RF and the ∆*zur* mutant grown for 1 hour in BHI, BHI with 30 µm TPEN, and BHI with 4 mM ZnSo_4_. Data points represent the average of six biological replicates. Statistical analysis was performed using the unpaired *t*-test with Welch’s correction. * *p* ≤.05. Error bars represent the standard error of margin (SEM).

Next, we used inductively coupled optical emission spectrometry (ICP-OES) to determine intracellular Zn pools in mid-log grown cultures of WT and derivative ∆*adc* strains grown in BHI or in BHI supplemented with 7.5 µM TPEN (Figure 3a). In BHI, the ∆*adcACB* accumulated less Zn when compared to the WT strain albeit this difference was rather small (~12%) and not supported by a similar or greater decrease in Zn pools in the ∆*adcA*∆*adcAII* and ∆*adcACB*∆*adcAII* strains (Figure 3a). In agreement with the predicted role of Zur as a transcriptional repressor of *adcABC* and *adcAII*, the ∆*zur* strain accumulated two times more Zn than the WT strain when grown in BHI. The addition of TPEN to the growth media led to an unexpected increase in intracellular Zn pools in the WT strain when compared to cells grown in BHI (~50% increase). Nonetheless, all mutants accumulated less Zn when compared to the WT strain when grown in BHI+TPEN. We suspected that the higher intracellular levels of Zn in the WT strain grown in BHI+TPEN compared to BHI only correlated with increased transcription of the *adcA*CB and *adcAII* genes and that this response was controlled by Zur. To verify this possibility, we used quantitative RT-PCR to determine mRNA levels of *adcA* and *adcAII* in the WT and ∆*zur* strains grown to mid-log phase in BHI and then treated with either 30 µM TPEN or 4 mM ZnSO_4_ for 1 h. In line with previous transcriptional studies [40,41], TPEN treatment significantly induced *adcA* (~1-log) and *adcAII* (~2-log) transcription, whereas Zn supplementation reduced *adcA* levels by ~2-log and *adcAII* by ~1-log when compared to the BHI control (Figure 3b). As expected, inactivation of *zur* resulted in increased transcription of *adcA* and *adcAII* grown in BHI (~1-log *adcA*, ~2-log *adcAII*) or BHI+Zn (~2-log both genes) when compared to the WT strain grown under the same conditions. Taken together, these results confirm that Zn deprivation (TPEN-treated cells) alleviates Zur repression triggering a strong induction of *adcACB* and *adcAII* expression that allows *E. faecalis* overcome Zn starvation.

### *AdcACB and AdcAII contribute to growth in serum but not in urine* ex vivo

**Figure 4.**
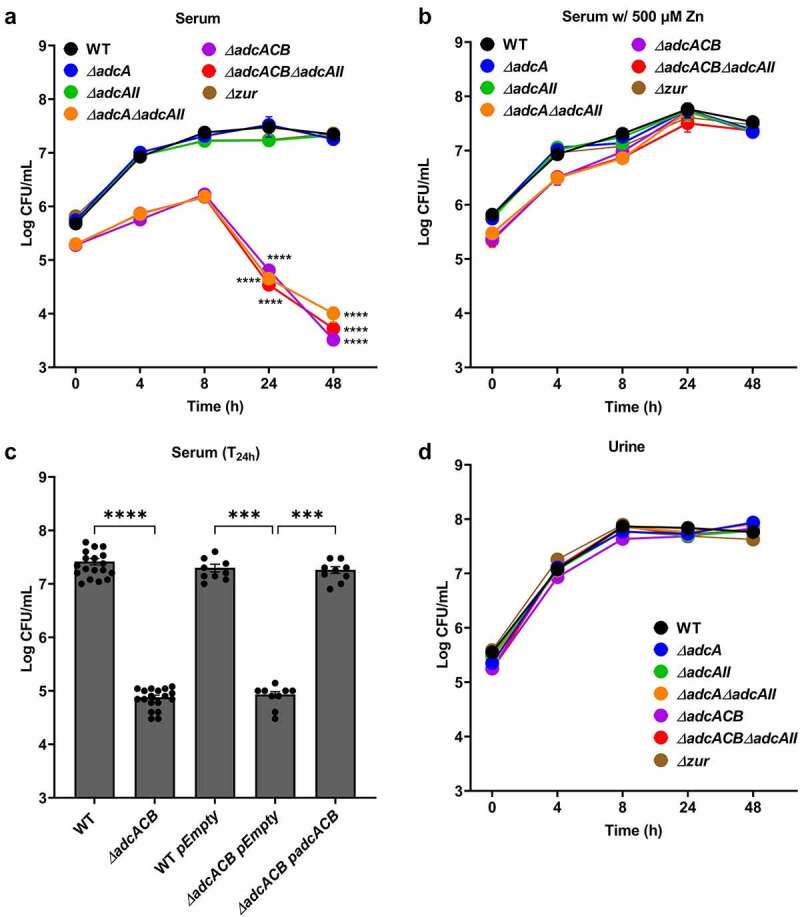
Growth and survival of *E. faecalis* in serum and urine. Colony-forming units (CFU) of *E. faecalis* OG1RF WT and its mutants incubated in pooled (a) human serum or (b) serum supplemented with 500 µm ZnSo_4_ and pooled human urine (d). (c) CFU counts of *E. faecalis* OG1RF WT, its mutants, and genetic complemented mutants after 24 hours of incubation in pooled human serum. In (a-d), data points represent the average and error bar represents the standard error of margin (SEM) of nine biological replicates. Statistical analysis was performed using one-way ANOVA with Welch’s correction. *** *p* ≤0.001 and **** *p* ≤0.0001.

To determine the contribution of Adc-mediated Zn uptake to *E. faecalis* virulence, we first monitored the ability of the ∆*adc* strains to grow and survive in pooled human serum or human urine *ex vivo*. When incubated in serum, growth of ∆*adcA* and ∆*adcAII* strains did not significantly differ from WT, whereas ∆*adcA*∆*adcAII*, ∆*adcACB* and ∆*adcACB*∆*adcAII* grew poorly and, most relevantly, displayed sharp decreases in survival after 8 hours and onward, ultimately showing a ~3-log reduction in colony-forming unit (CFU) recovered after 48 hours of incubation in serum (Figure 4a). These growth and survival defects were fully reversed by the addition of 500 µM ZnSO_4_ (Figure 4b) or *in trans* complementation (Figure 4c). On the other hand, the ability of all ∆*adc* strains to grow/survive in urine was not found to differ from WT (Figure 4d), suggesting that Zn is not a growth-limiting factor in urine (at least *ex vivo*). Finally, the inactivation of *zur* did not impact growth nor survival in serum or urine (**Figure 4a-b,d)**.

While Zn levels and bioavailability in serum or in urine were not determined, the strong phenotype of the ∆*adcA*∆*adcAII*, ∆*adcACB* and ∆*adcACB*∆*adcA* strains in serum was expected as Zn levels in blood circulation are low with most Zn sources bound or sequestered by host cells and proteins [45,46]. On the other hand, Zn is abundant in the bladder environment as any excess Zn, typically from dietary sources, is excreted through urine via the gastrointestinal route [47–50].

### Disruption of AdcABC-AdcAII lowers tolerance toward cell envelope-targeting antibiotics

**Figure 5.**
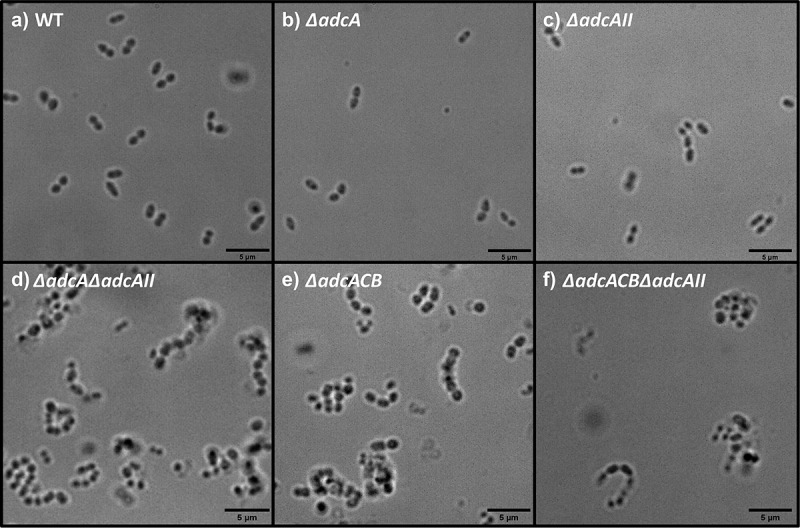
Bright-field microscopic images of *E. faecalis* and its indicated mutants. Images shown are representative of 10 images that are acquired from one biological sample from each strain grown in BHI, respectively, and imaged at 100x magnification. Black bars represent five microns in length.

**Figure 6.**
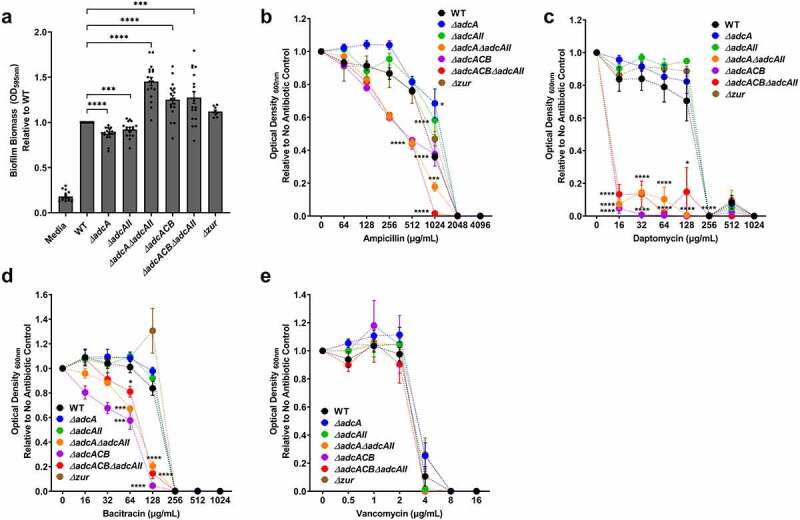
Characterization of *E. faecalis* virulence traits at the cell surface interface. (a) Biofilm biomass quantification of *E. faecalis* OG1RF WT and its mutants grown in BHI for 24 hours. Statistical analysis was performed using One-way ANOVA with Welch’s correction. (b-e) Final growth yields of *E. faecalis* OG1RF WT and its mutants after 24 hours of incubation in BHI supplemented with 2-fold increasing concentrations of (b) ampicillin, (c) daptomycin, (d) bacitracin, and (e) vancomycin. Data points represent the average of nine biological replicates. Statistical analysis was performed using One-way ANOVA with Welch’s correction. * *p* ≤0.05, *** *p* ≤0.001, and **** *p* ≤0.0001. Error bars represent the standard error of margin (SEM).

Because the ∆*adcA*∆*adcAII*, ∆*adcACB* and ∆*adcACB*∆*adcAII* strains displayed a reduced growth rate under Zn-restricted conditions, like *S. pneumoniae* Δ*adcA*Δ*adcAII* [16], we wondered if this was due to altered cell division. To investigate this, we observed bacterial morphology using a light microscope. Indeed, these mutants formed longer chains when compared to the WT, ∆*adcA* and ∆*adcAII* strains that primarily formed only short chains or diplococcus (Figure 5). This observation and the fact that *S. pneumoniae* Δ*adcA*Δ*adcAII* mutant displayed aberrant cell septation [16] led us to wonder if expression of virulence traits that occur at the cell surface interface were similarly affected in the mutant strains. First, we compared the capacity of WT and mutants to form biofilms after 24 hours of incubation in BHI supplemented with 10 mM glucose. The total biofilm biomass of ∆*adcA* and ∆*adcAII* single mutants was significantly reduced when compared to WT, but the very small differences observed (5 to 10% reduction) are unlikely to have major biological implications (Figure 6a). On the other hand, the ∆*adcA*∆*adcAII*, ∆*adcACB* and ∆*adcACB*∆*adcAII* strains formed more robust biofilms with an ~30 to 50% increase in biofilm biomass (Figure 6a).

Next, we tested the capacity of WT and mutants to grow in BHI supplemented with antibiotics that target different steps of cell wall biosynthesis (ampicillin, bacitracin, and vancomycin) or disrupt membrane integrity (daptomycin) by determining the minimal inhibitory concentration (MIC) for each antibiotic. Once again, relevant phenotypes were restricted to the ∆*adcA*∆*adcAII*, ∆*adcACB*, and ∆*adcACB*∆*adcAII* strains. Specifically, ∆*adcA*∆*adcAII*, ∆*adcACB*, and ∆*adcACB*∆*adcAII* showed lower MICs for ampicillin, bacitracin, and daptomycin (Figure 6b-d). However, WT and all mutants showed the same MIC for vancomycin (Figure 6e).

### *Zn is critical for* E. faecalis *virulence during infection*

**Figure 7.**
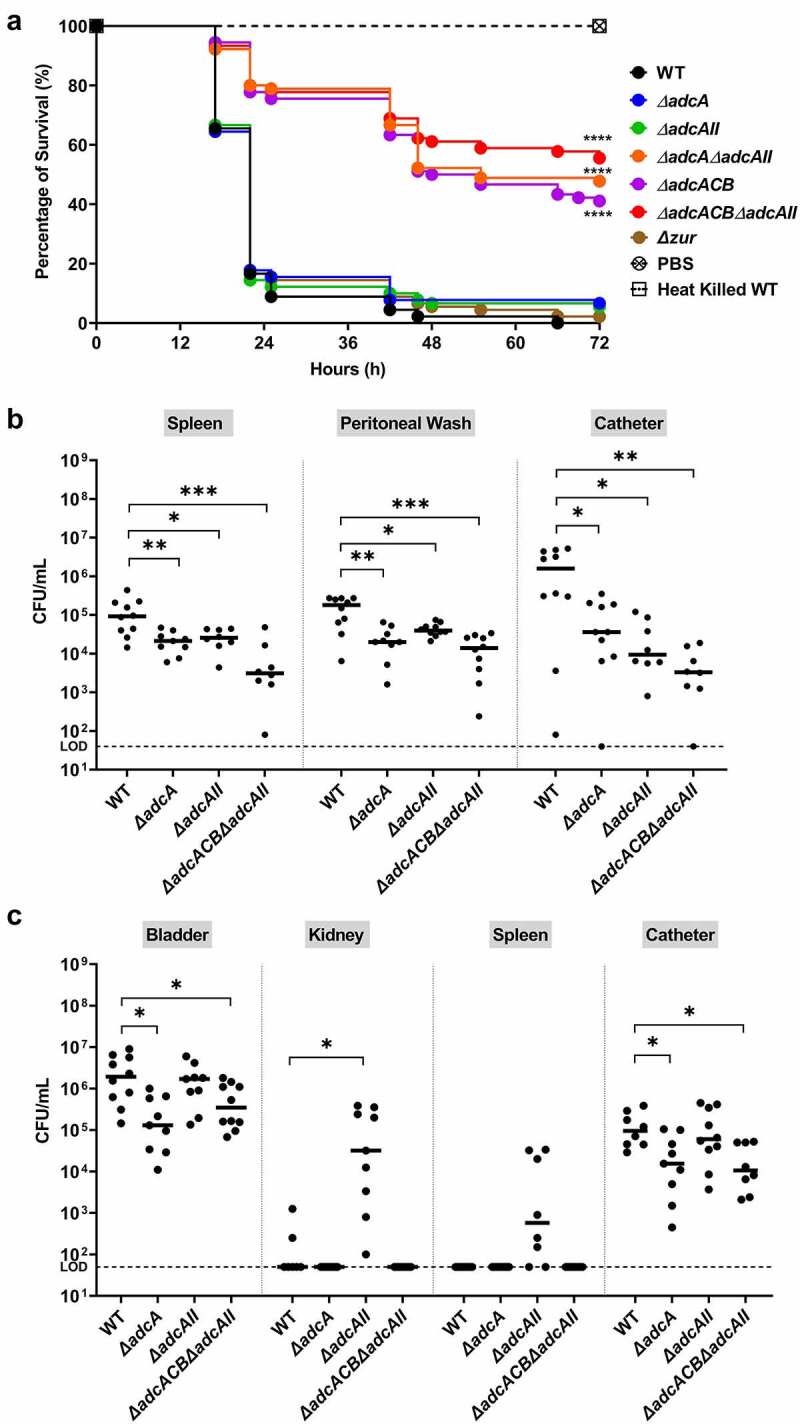
Virulence of *E. faecalis* in different animal models. (a) *P*ercentage survival of *G. mellonella* larvae 96 hours post-infection with *E. faecalis* WT or indicated mutants. Each curve represents a group of 15 larvae injected with ~1 x 10^5^ CFU of selected *E. faecalis* strain. Data points represent the average of 6 biological replicates. Statistical analysis was performed using the log-rank (Mantel-Cox) test. (b) Total CFU recovered after 48 hours from spleen, peritoneal wash, and catheter of mice infected with 2 × 10^8^ CFU of bacteria. (c) Total CFU recovered after 24 hours from bladder, kidney, spleen, and catheter of mice infected with 1 × 10^7^ CFU of WT or indicated mutants. In (b and c), ten mice were infected with two biological replicates and data points shown were a result of using the ROUT outlier test. The black line represents the median. Statistical analysis was performed using the Mann-Whitney test. * *p* ≤0.05, ** *p* ≤0.01, *** *p* ≤0.001, and **** *p* ≤0.0001. The dashed line represents the limit of detection (LOD = 50 CFUs).

In the last series of experiments, we used three *in vivo* models to probe the contributions of AdcABC and AdcAII to virulence. In conformity with *in vitro* and *ex vivo* phenotypes, virulence of ∆*adcA*∆*adcAII*, ∆*adcACB* and ∆*adcACB*∆*adcAII* but not the single mutants (∆*adcA*, ∆*adcAII* and ∆*zur*) was highly attenuated in the *Galleria mellonella* model (Figure 7a). Next, we used two catheter-associated mouse infection models that recapitulate some of the environmental and immunological conditions that promote enterococcal infections in human. From this point, we did not include the ∆*adcA*∆*adcAII* and ∆*adcACB* strains as these mutants consistently phenocopied the ∆*adcACB*∆*adcAII* mutant. In the catheter-associated peritonitis model, the ∆*adcA* and ∆*adcAII* single mutants colonized the peritoneal cavity, catheter and spleen (infection becomes systemic after 12 to 24 h) in significantly fewer numbers than the WT strain (Figure 7b). Not surprisingly, colonization defects of single mutants were significantly more pronounced in the ∆*adcACB*∆*adcAII* strain. Finally, in a catheter-associated urinary tract infection (CAUTI) model, virulence of ∆*adcA* and ∆*adcACB*∆*adcAII* strains was attenuated, but bacterial burden recovered from bladders of retrieved catheter of animals infected with WT or ∆*adcAII* strains were nearly identical (Figure 7c). Moreover, ∆*adcAII* was recovered from kidneys and spleen in higher bacterial titers, albeit the trend of the latter was not considered significant (Figure 7c).

## Discussion

While there has been increasing appreciation of the multiple contributions of trace metals other than Fe to bacterial fitness and virulence [2,5,20], the mechanisms utilized by *E. faecalis* to maintain Zn homeostasis and their specific contributions to pathogenesis were, until now, poorly understood. Previously, our group showed that *E. faecalis* encodes three high-affinity Mn transporters and that while it was necessary to inactivate all three Mn transport systems (*efaCBA*, *mntH1* and *mnthH2*) to severely impair Mn uptake *in vitro*, the inactivation of only two of them (*efaCBA* and *mntH2*) was sufficient to abolish *E. faecalis* virulence [51]. In this report, we showed that both AdcACB and the orphan AdcAII predicted to mediate Zn import in a Zur-regulated manner and are critical for the ability of *E. faecalis* to grow and survive Zn-restricted conditions. Although the differences were not as striking as those seen with the Mn transport mutants [51], virulence of strains lacking the AdcACB/AdcAII system was significantly attenuated in both invertebrate and vertebrate infection models.

During characterization of the *adc* mutants, we noted that simultaneous deletion of *adcACB* and *adcAII* led to readily discernible morphological and biophysical alterations, that is increased cell chaining and cell-cell aggregation, which led us to wonder if the inability to maintain Zn homeostasis affected cell envelope homeostasis. Indeed, the ∆*adcA*∆*adcAII*, ∆*adcACB*, and ∆*adcACB*∆*adcAII* strains showed heightened sensitivity to ampicillin, bacitracin, and daptomycin, with the latter showing a striking 16-fold lower MIC than the WT strain MIC (≤16 μg ml^−1^ml, compared to 256 μg ml^−1^). Previous studies have shown that exposure to bacitracin resulted in downregulation of *adcA* (0.2-fold) in *E. faecalis* V583, whereas *adcAII* transcription increased (8-fold) after vancomycin treatment [52]. In addition, exposure to chlorhexidine, a cationic antimicrobial agent that targets the cell membrane, resulted in upregulation of *adcCB* (~4-fold) [53]. While the roles played by Zn in *E. faecalis* envelope homeostasis are unknown, previous studies have associated loss of Zn transporters or Zn-dependent enzymes to surface-associated defective phenotypes. For example, in *S. pneumoniae*, deletion of *adcACB* and *adcAII* resulted in asymmetrical septa formation, abnormal cell division patterns, and emergence of small, aborted cells when *S. pneumoniae* was forced to grown under Zn-restricted conditions [16]. In the distantly related Gram-negative pathogen *Acinetobacter baumannii*, inactivation of a Zn-dependent peptidase, ZrlA, increased cell permeability and susceptibility to the β-lactam antibiotic carbenicillin [54].

Similar to streptococci, enterococcal genomes do not encode the machinery to synthesize opine-like zincophores and their cognate transporters. In Gram-positive cocci, Zn acquisition is primarily mediated by *adcABC* (also known as *znuABC*) and *adcAII*, both of which are under AdcR (ZuR) negative control [16,22,26,29,55]. Similar to our findings showing the additive contribution of *E. faecalis adcA* and *adcAII* genes to Zn homeostasis, simultaneous inactivation of both *adcA* and *adcAII* is necessary to (nearly) abolish Zn import and, as a result, drastically impairs virulence of major human pathogens such as *S. pyogenes* and *S. pneumoniae* [16,26,27].

Although *E. faecalis* AdcA and AdcAII share 52% identity, they do not have a similar domain organization. Biochemical and biophysical characterizations of *S. pneumoniae* AdcA and AdcAII proteins indicated that these functionally redundant proteins employ distinct Zn acquisition mechanisms [17]. AdcAII_*Spn*_ has two Zn-binding domains, an amino terminal cluster A-I domain typical of solute-binding proteins and a C-terminal domain that is structurally related to ZinT, a periplasmic Zn chaperone of Gram-negative bacteria, whereas AdcA_*Spn*_ has only the terminal cluster A-I domain [17]. Moreover, AdcAII_*Spn*_-mediated Zn uptake *in vivo* has been shown to depend on proteins with poly-histidine (Pht) triad (HxxHxH) motifs that scavenge extracellular Zn and then transfer it to AdcAII_*Spn*_ for internalization [56]. While streptococcal species have been shown to encode as many as four Pht protein homologues (e.g., *S. pneumoniae* PhtA, PhtB, PhtD and PhtE), *E. faecalis* genomes do not contain genes with HxxHxH motifs. Of interest, a small open-reading frame (*OG1RF_RS12620*) (40 amino acids) coding for a putative uncharacterized protein is located upstream and separated by only 21-bp from the *adcAII*_*Ef*_ start codon. While the predicted amino acid sequence of the *OG1RF_RS12620* contains only a single histidine residue and is not enriched for other amino acids (such as cysteine and methionine) that typically coordinate Zn, it will be interesting to explore the possible role of *OG1RF_RS12620* in Zn acquisition in future studies.

Because an *E. faecalis* strain lacking the entire *adcACB* operon is phenotypically similar to the *∆adcA∆adcAII* double mutant, an important observation from this study is that, most likely, AdcA and AdcAII can only form functional complexes with AdcB (inner membrane permease) and AdcC (cytoplasmic ATPase). Also of interest was the distinct importance of AdcA and AdcAII to colonization and systemic dissemination in the two mouse models. While attenuated virulence of *∆adcA* and *∆adcAII* single mutants was comparable in the peritonitis model, *adcAII* was dispensable for bladder and catheter colonization in the CAUTI model. In addition, the recovery of viable bacteria from kidney and spleen of mice infected with ∆*adcAII* indicates, at first glance, that the loss of AdcAII promotes bacterial dissemination to kidneys and spleen. While speculative at this point, we believe that these phenotypes are due to differences in the expression levels or activity of AdcA and AdcAII in the bladder environment. One possibility is that *adcA* responds more strongly than *adcAII* to environmental cues encountered in the bladder, such that *adcAII* becomes dispensable for *E. faecalis* proliferation in urine. To test this possibility, studies to compare the *adc* transcriptional profiles of ∆*adcA* and ∆*adcAII* in the bladder, peritoneal cavity, and bloodstream environments will soon be underway. Alternatively, the large and constant fluctuations in the bladder environment in solute concentrations and of other important biophysical and biochemical parameters such as pH caused by intermittent cycles of urination can somehow compromise Zn-binding capacity of AdcAII or ability to interact with the AdcBC partner proteins.

In summary, our findings reveal that the AdcACB/AdcAII system is a bona fide Zn acquisition system of *E. faecalis* contributing additively to the maintenance of Zn homeostasis. More importantly, this report demonstrates that the AdcACB/AdcAII system mediates *E. faecalis* virulence. Therefore, both the substrate-binding and surface-associated AdcA and AdcAII proteins can be viewed as suitable targets for the development of antimicrobial therapies to treat or prevent enterococcal infections.

## Material and methods

### Bacterial strains and growth conditions

The bacterial strains and vectors used in this study are listed in Table 1. Bacteria were routinely grown in brain heart infusion (BHI broth (BD Difco^TM^, for *E. faecalis*) and Luria-bertani (BD Difco^TM^, for *E. coli*) at 37°C under static conditions. Strains possessing the pGCP123 plasmid [59] were grown in the presence of kanamycin (300 µg ml^−1^ for *E. coli* and 500 µg ml^−1^ for *E. faecalis*). For growth kinetics assays, overnight cultures were normalized by cell density to an OD_600_ of 0.25 and inoculated into BHI media at a 1:50 ratio, with the OD_600_ monitored in an automated growth reader (Bioscreen c, Oy Growth Curves AB). Native calprotectin (hCP) and Mn-deficient (Cp ^∆Mn-^^tail^) calprotectin were gifts from Dr. Walter Chazin (Vanderbilt University, USA) [10]. Experiments using purified calprotectin were performed in BHI supplemented with 20% (v/v) CP buffer (40 mM NaCl, 0.5 mM β-mercapethanol, 1.2 mM CaCl_2_, 8 mM Tris-HCl, pH 7.5). Normalization of starter cultures for most experiments included centrifugation or overnight cultures at 4000 rpm for 10 mins to remove spent media, two cell pellet washes in PBS, and adjustment of cell density to an OD_600_ of 0.25 (~1 × 10^8^ CFU ml^−1^) also in PBS. For *E. faecalis* CFU determination from *ex vivo* and *in vivo* studies, serially diluted aliquots were plated on BHI agar supplemented with 200 µg ml^−1^ rifampicin and 10 µg ml^−1^ fusidic acid. TPEN (N,N,N′,N′-tetrakis(2-pyridinylmethyl)-1,2-ethanediamine), FeSO_4_, MnSO_4_, ZnSO_4_, lysozyme, ampicillin, bacitracin, daptomycin, fusidic acid, kanamycin, rifampicin, and vancomycin were purchased from Sigma Aldrich.

**Table 1.** Strains and plasmids used in this study

	Strain name	Relevant characteristics	Plasmid	References
*E. faecalis* parent strain	OG1RF wild type (WT)	Laboratory strain, Rif*R*, Fus^R^	-	[57]
*E. faecalis* deletion mutants	OG1RF ∆*adcA*	*OG1RF_RS00260* deletion	-	This study
	OG1RF ∆*adcA-II*	*OG1RF_RS12625* deletion	-	This study
	OG1RF ∆*adcA*∆*adcA-II*	*OG1RF_RS00260* and *OG1RF_RS12625* deletion	-	This study
	OG1RF ∆*adcACB*	*OG1RF_RS00260-70* deletion	-	This study
	OG1RF ∆*adcACB*∆*adcA-II*	*OG1RF_RS00260-70* and *OG1RF_RS12625* deletion	-	This study
	OG1RF ∆*zur*	*OG1RF_RS09465* deletion	-	This study
*E. faecalis* conjugative strains and plasmid	CK111	OG1Sp *upp4*::P23*repA4*, Spec*R*	pCJK47	[42]
	CK111	OG1Sp *upp4*::P23*repA4*, Spec*R*	pCJK47:*adcA-II*	This study
	CK111	OG1Sp *upp4*::P23*repA4*, Spec*R*	pCJK47:*adcA*	This study
	CK111	OG1Sp *upp4*::P23*repA4*, Spec*R*	*p*cJK47:*zur*	This study
	CK111	OG1Sp *upp4*::P23*repA4*, Spec*R*	pCJK47:*adcCB*	This study
*E. faecalis* complementation strains	OG1RF gcp123	Empty vector, Kan*R*	gcp123	This study
	OG1RF ∆*adcACB* gcp123	Empty vector, Kan*R*	gcp123	This study
	OG1RF ∆*adcACB* gcp123:*adcACB*	Vector contains *OG1RF_RS00260* coding sequence and putative native promoter sequence (250 bps upstream), Kan*R*	gcp123:*adcACB*	This study
	OG1RF ∆*adcA-II* gcp123	Empty vector, Kan*R*	gcp123	This study
	OG1RF ∆*adcA-II* gcp123:*adcA-II*	Vector contains *adcA-II* coding sequence and *adcA* native promoter sequence (250 bps upstream), Kan*R*	gcp123:*adcA-II*	This study
*E. coli* strains for cloning	EC1000	Host and carrier for RepA-dependent cloning using pCJK47 vector, carries pheS*; Erm*R*	*p*cJK47[42]	[42,58]
	Stellar *E. coli*	Cloning host (Takara Bio)	-	-

### Homology searches and structural modeling of proteins using BlastP, AlphaFold, and Chimera

Amino acid sequences from AdcA and AdcAII proteins of *E. faecalis* OG1RF (AdcA; WP_002367576.1, AdcAII; WP_002392710.1), *S. pneumoniae* R6 (AdcA; WP_000724074.1, AdcAII; WP_001844050.1), and *S. pyogenes* MGAS5005 (AdcA; WP_011285462.1, AdcAII; WP_002987954.1) were queried for conservation of sequences using NCBI BlastP multiple sequence alignment to generate an alignment highlighting consensus sequence. For structural modeling, the amino acid sequences of *E. faecalis* OG1RF AdcA and AdcAII and *S. pneumoniae* R6 AdcA and AdcAII were retrieved from PubMed NCBI database. Tertiary structures were obtained using AlphaFold Colab notebook [60,61], and image files (PDB) were constructed using ChimeraX1.3 [62].

### General cloning techniques

Bacterial genomic DNA (gDNA) was isolated using a Wizard Genomic DNA purification kit (Promega). Plasmid purification was performed using the Monarch plasmid miniprep kit (New England BioLabs). Isolation of PCR amplified products was performed using the Monarch DNA gel extraction kit (New England BioLabs). Typical cloning, either directional or non-directional depending on plasmid used, was performed using the In-Fusion HD cloning kit (TaKaRa Bio). Colony PCR was performed using PCR 2x Master Mix (Promega) with primers listed in Table 2.

**Table 2.** Primers used in this study

	Primer name	Primer sequence (5’ → 3’)	Restriction sites^a^
Primers for qRT-PCR	adcA_F’	TCGTGGAAGCCAGTCAATCC	-
	adcA_R’	TTTGCGCTAACACGGGATCT	-
	adcA-II_F’	TTCTGGTCGATGGCAAACCA	-
	adcA-II_R’	GTTGGTGCGATGTTGTGGTC	-
Primers for cloning in pCJK47 vector	adcA_Arm1 F’	TCGCTAGTTCTAGA**GCGGCCGC**CTTATTAAAGCGTACCTATAATATT	NotI
	adcA_Arm1 R’	TGTTGCCAGAGTTCTGTCCTCTTTTCGATTT	-
	adcA_Arm2 F’	AGGACAGAACTCTGGCAACAGAAAGGAGCCAA	-
	adcA_Arm2 R’	CTTAGCATGCCATGGTA**CCCGGG**TTTTTTAAATTTTTCCCACATTTCT	SmaI
	adcA-II_Arm1 F’	TCGCTAGTTCTAGA**GCGGCCGC**ATCAAAAATCATTTCTATATAGACA	NotI
	adcA-II_Arm1 R’	TTTTGAATGCAGGTGTGCTCCTTTAGTG	-
	adcA-II_Arm2 F’	GAGCACACCTGCATTCAAAAGAGAGGAGGTAGCA	-
	adcA-II_Arm2 R’	CTTAGCATGCCATGGTA**CCCGGG**GGGATAAACCGGAATTTTTCCGTGA	SmaI
	adcCB_Arm1 F’	TCGCTAGTTCTAGA**GCGGCCGC**TCATGTCTATCAAGCAGTTGAC	NotI
	adcCB_Arm1 R’	GTTTTTCTCCGTTCTGTCCTCTTTTCGATTTACC	-
	adcCB_Arm2 F’	AGGACAGAACGGAGAAAAACGAAAGTTTTTCTCCT	-
	adcCB_Arm2 R’	GGAAGACTTGGCAGATTGGTATCGGTCAGCA	-
	adcCB_Arm3 F’	ACCAATCTGCCAAGTCTTCCATTAGTGAAGA	-
	adcCB_Arm3 R’	ACACAGCTTGCGCAATTGGCACTCCTTT	-
	adcCB_Arm4 F’	GCCAATTGCGCAAGCTGTGTCTTATTACTTAAATG	-
	adcCB_Arm4 R’	CTTAGCATGCCATGGTA**CCCGGG**CATTGAATTTTGACTCAGCAAAAAC	SmaI
	zur_Arm1 F’	TCGCTAGT**TCTAGA**GCGGCCTGTTTCCGTGGAATAATAACTATGA	XbaI
	zur_Arm1 R’	GTCTTATTCGCGCCATTTCCTCCCTCAC	-
	zur_Arm2 F’	GGAAATGGCGCGAATAAGACGAAAAATCAAACAAA	-
	zur_Arm2 R’	CTTA**GCATGC**CATGGTACCCCCACATTCTTATCTGTTTAAAAAGA	SphI
	*p*cJK47 F’	GATTTCAGAATCGCTAGTTCTAGA	-
	*p*cJK47 R’	ATGTATTCACGAACGAAAATCAAG	-
Primers for screening deletion mutants	adcA_F’	TTGACGTCCCGTGATCCATT	-
	adcA_R’	AGCGACCGTAATTTTGCCAC	-
	adcA-II_F’	AACTGACTTATCTAGCGCTTATCGT	-
	adcA-II_R’	AGCGACGTTTGAAGCAGAAC	-
	adcACB_F’	ACTTTGGGTTTTTGCTCCACG	-
	adcACB_R’	ATCGTTCACTTCTCCGAATTTTCA	-
	zur_F’	CGCCAATTTTGAAGCGGTCT	-
	zur_R’	ATTCCCTCCCTCCGAGCTTA	-
Primers for cloning in gcp123 vector	gcp123 F’	GTAAAACGACGGCCAGTGAGC	-
	gcp123 R’	CCAGGAAACAGCTATGACCATGAT	-
	adcA-II Arm1 F’	GATAAGCTTGATATCGAATTCGACAGTCTTTGCTTTATGTGA	-
	adcA-II Arm1 R’	ATTTTTTCATGTTCTGTCCTCTTTTCGATTTACCT	-
	adcA-II Arm2 F’	AGGACAGAACATGAAAAAATTTACTCTTCCCCTGT	-
	adcA-II Arm2 R’	CGCTCTAGAACTAGTGGATCTTAATGCGCAATCATTTCTTGGG	-
	adcACB Arm1 F’	GATAAGCTTGATATCGAATTCGACAGTCTTTGCTTTATG	-
	adcACB Arm1 R’	TGTTGCCAGATTAATGAATGCTTTTTTGTAAAG	-
	adcACB Arm2 F’	CATTCATTAATCTGGCAACAGAAAGGAGCCA	-
	adcACB Arm2 R’	TTTCTGCCATACGATTCACTCCTTACCGCT	-
	adcACB Arm3 F’	AGTGAATCGTATGGCAGAAATGCTTTCTTATGCA	-
	adcACB Arm3 R’	CGCTCTAGAACTAGTGGATCTTAGTTTCTTTGCATTTTTTGTTTT	-

^a^Restriction sites are underlined and bold in the primer sequence.

### Construction of deletion and genetically complemented strains

Deletion of *zur*, *adcA*, *adcAII*, or the entire *adcACB* operon was carried out using a markerless genetic exchange system based on the pCJK47 vector [42]. Briefly, nucleotide sequences flanking *zur*, *adcA, adcCB*, and *adcAII* were amplified using the primers listed in Table 2. These PCR-amplified products were directly cloned into pcJK47, electroporated into *E. faecalis* CK111 (donor strain), followed by conjugation into *E*. *faecalis* OG1RF. The deletion mutants were isolated by following the steps for markerless counterselection detailed elsewhere [42]. The ∆*adcA*∆*adcAII* and ∆*adcACB*∆*adcAII* double mutants were obtained by conjugating the pCJK-*adcAII* plasmid with the ∆*adcA* and ∆*adcACB* mutants, respectively. Because the *adcACB* operon is ~3kb, we first isolated ∆*adcA* and then performed conjugation using the pCJK-*adcBC* vector to generate the ∆*adcACB* strain. PCR sequencing was performed to confirm the absence of *zur*, *adcA*, *adcAII*, or the entire *adcACB* operon in these deletion mutant strains. For genetic complementation, *adcACB* and *adcAII* genes were amplified by PCR and cloned into the pGCP123 plasmid [59] using the primers listed in Table 2. The full-length *adcACB* and *adcAII* nucleotide sequences were incorporated into linearized pGCP123 plasmid using the In-Fusion HD cloning kit. Plasmids were propagated in *E. coli* and transformed into *E. faecalis* as described previously [59].

### Inductively coupled plasma-optical emission spectrometry (ICP-OES)

The metal (Zn) concentration in BHI media and in bacteria was determined as previously described [51]. For quantification of metals in BHI, 9 ml of broth was digested with 1 ml of trace-metal grade 35% nitric acid (HNO_3_) prior to analysis. For quantification of intracellular metal, overnight *E. faecalis* cultures were washed with PBS twice and inoculated at a ratio of 1:40 in BHI or BHI supplemented with 7.5 µM TPEN. Mid-log grown cultures (OD_600_ of 0.5) were harvested by centrifugation and washed twice with PBS containing 0.5 mM EDTA. After washing, cell pellets were collected in a polyethylene scintillation vial (Fisher Scientific) and digested in 1 ml of trace-metal grade 35% nitric acid (HNO_3_) at 90°C for 1 hour. Digested bacterial cells were diluted at a ratio of 1:10 in reagent-grade water prior to analysis. Intracellular Zn pools were then quantified using a 5300DV ICP Atomic Emission Spectrometer (Perkin Elmer) at the University of Florida Institute of Food and Agricultural Sciences (UF-IFAS) Analytical Services Laboratories. The bicinchoninic acid (BCA) assay kit (Pierce^TM^) was used to calculate the total protein content for normalization of the metal concentration obtained.

### MIC determinations

Overnight *E. faecalis* cultures were normalized to an OD_600_ of 0.25 and diluted at a ratio of 1:1000. The diluted cultures were inoculated at a ratio of 1:20 into BHI containing antibiotics (ampicillin, bacitracin, daptomycin, and vancomycin). The absorbance at OD_600_ after incubation at 37°C for 24 hours was measured using a Synergy H1 microplate reader (Molecular Devices).

### Growth and survival in serum and urine

Inoculum was prepared from overnight *E. faecalis* cultures adjusted to OD_600_ of 0.5 and inoculated into pooled human serum or human urine (Lee Biosolutions) at a ratio of 1:1000. Aliquots were obtained at several time points and then plated on BHI agar containing fusidic acid and rifampicin to determine CFU.

### Biofilm assay

Inoculum was prepared from overnight *E. faecalis* cultures adjusted to OD_600_ of 0.5 that were diluted 1:25 in BHI supplemented with 10 mM glucose. Biofilm assay was performed using 96-well polystyrene plates (Grenier) that were incubated at 37°C for 24 hours. Post-incubation, spent media was discarded, biofilms washed twice with PBS, and the biomass stained with 0.1% crystal violet for 25 mins. A 33% acetic acid solution was used to dissolve the precipitated crystal violet-stained biomass, and absorbance was determined at OD_595_.

### Quantitative real-time PCR

Overnight *E. faecalis* cultures were normalized to an OD_600_ of 0.5, inoculated at a ratio of 1:20 into fresh BHI, and incubated for 1 hour at 37°C. After incubation, cells were collected by centrifugation at 4000 rpm for 10 mins, washed with PBS, and incubated in the presence of lysozyme (20 mg ml^−1^) at 37°C for 30 mins. After treatment, cells were harvested by centrifugation and the total RNA was extracted using the PureLink RNA minikit (Invitrogen). Next, a Turbo DNA-free kit (Thermo Fisher) was used for purification of RNA and removal of contaminating gDNA. A High-capacity cDNA Reverse Transcription Kit (Applied Biosystems) was used for the synthesis of cDNA. Quantitative real-time PCR (qRT-PCR) was performed using iTaq Universal SYBR supermix (BioRad) with the primers listed in Table 2. For quantification of transcript numbers, *E. faecalis* OG1RF gDNA was used as a template to generate standard curves.

### Galleria mellonella *infection*

To assess *E. faecalis* virulence, the larvae of *G*. *mellonella* were used as described previously [51,63]. Briefly, larvae (groups of 15) were injected with exponentially grown cells (~5 × 10^5^ CFU), heat-killed *E*. *faecalis* (30 mins at 100°C negative control), or PBS (vehicle control). Post-injection, larvae were kept at 37°C and their survival were recorded over time.

### Catheter-Associated peritonitis mouse model

The methods for the catheter-associated peritonitis model have been described previously [64], and hence, only a brief overview describing minor modifications are described here. Female C57BL/6J 8-weeks old mice were purchased from Jackson Laboratories and allowed to acclimate for at least 2 days prior to initiation of the study. Three hours post-catheter implantation, mice were injected intra-peritoneally with ~5 × 10^8^ CFU of bacteria from an overnight culture. Forty eight hours post-injection, mice were euthanized and the bacterial burden was determined by plating serially diluted aliquots on selective BHI plates supplemented with rifampicin and fusidic acid. This procedure was approved and performed in compliance with the University of Florida Institutional Animal Care and Use Committee (protocol# 201910705).

### Mouse catheter-associated urinary tract infection (CAUTI) model

The full description of the method for the CAUTI infection model has also been described in detail previously [65], and hence, a brief overview and modifications are described here. Female C57BL/6Ncr 6-weeks old mice were purchased from Charles River Laboratories and subjected to transurethral catheter implantation. Post-catheter implantation, mice were infected with **~**1 × 10^7^ CFU of bacteria from an overnight culture. 24 hours post-infection, mice were euthanized, and bacterial burden were determined by plating on selective BHI plates supplemented with rifampicin and fusidic acid. This procedure follows the University of Notre Dame Institutional Animal Care and Use Committee (protocol #18 August 4792MD).

### Bright field microscopy

Overnight cultures grown in BHI were normalized to OD_600_ 0.5 (~5×10^8^ CFU/ml) and washed once with 1 ml of PBS. Aliquot of 5 µl of bacterial inoculum was placed on microscope glass sides that were washed once in filtered 70% ethanol followed by milliQ water and left to dry at room temperature. Samples were covered with 5 µl of mounting media (Vectashield) and covered with a glass coverslip. Bright field microscopy was performed using a Leica DM2500 LED optical microscope fitted with a 100X/1.3 oil objective lens. Images acquired were further processed using FIJI software [66].

### Statistical analysis

Data obtained from this study were analyzed using GraphPad Prism 9.0 software (GraphPad Software, San Diego, CA, USA). Data from multiple experiments conducted on nonconsecutive days were collated, and applicable statistical tests were used.

## Supplementary Material

Supplemental Material

## Data Availability

The authors confirm that the data supporting the findings of this study are available within the article and/or its supplementary materials. Vectors and strains created from this study will be available from the corresponding author upon reasonable request.
