# Supplementary figures and images for: The AdcACB/AdcAII system is essential for zinc homeostasis and an important contributor of Enterococcus faecalis virulence
Source: Virulence. 2022 Mar 28;13(1):592–608. doi: 10.1080/21505594.2022.2056965 (PMC8966984; doi:10.1080/21505594.2022.2056965)

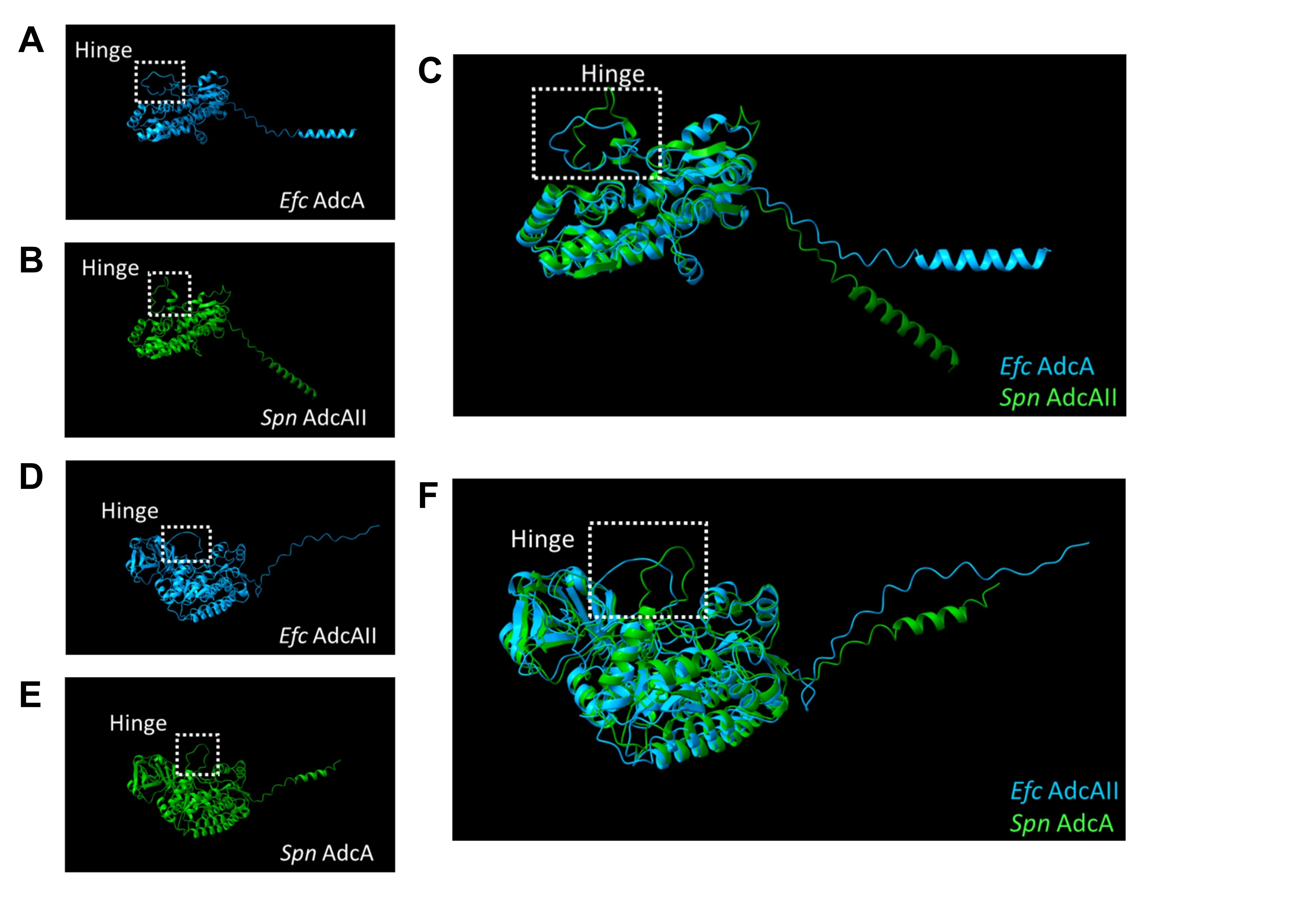

Supplement: Supplemental Material [file KVIR_A_2056965_SM5462.zip › supplementary/Fig S2.jpg]

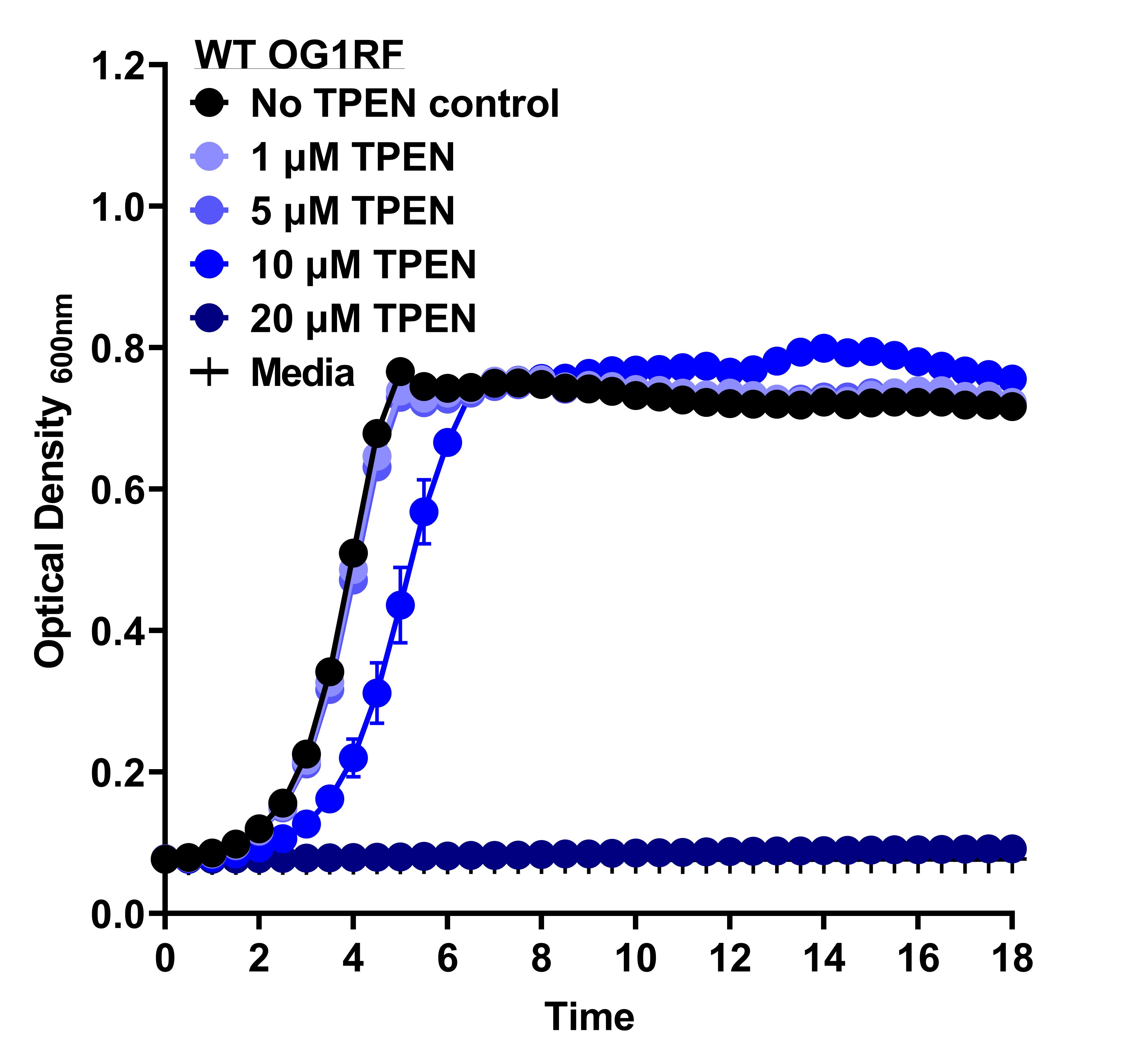

Supplement: Supplemental Material [file KVIR_A_2056965_SM5462.zip › supplementary/Fig S3.jpg]

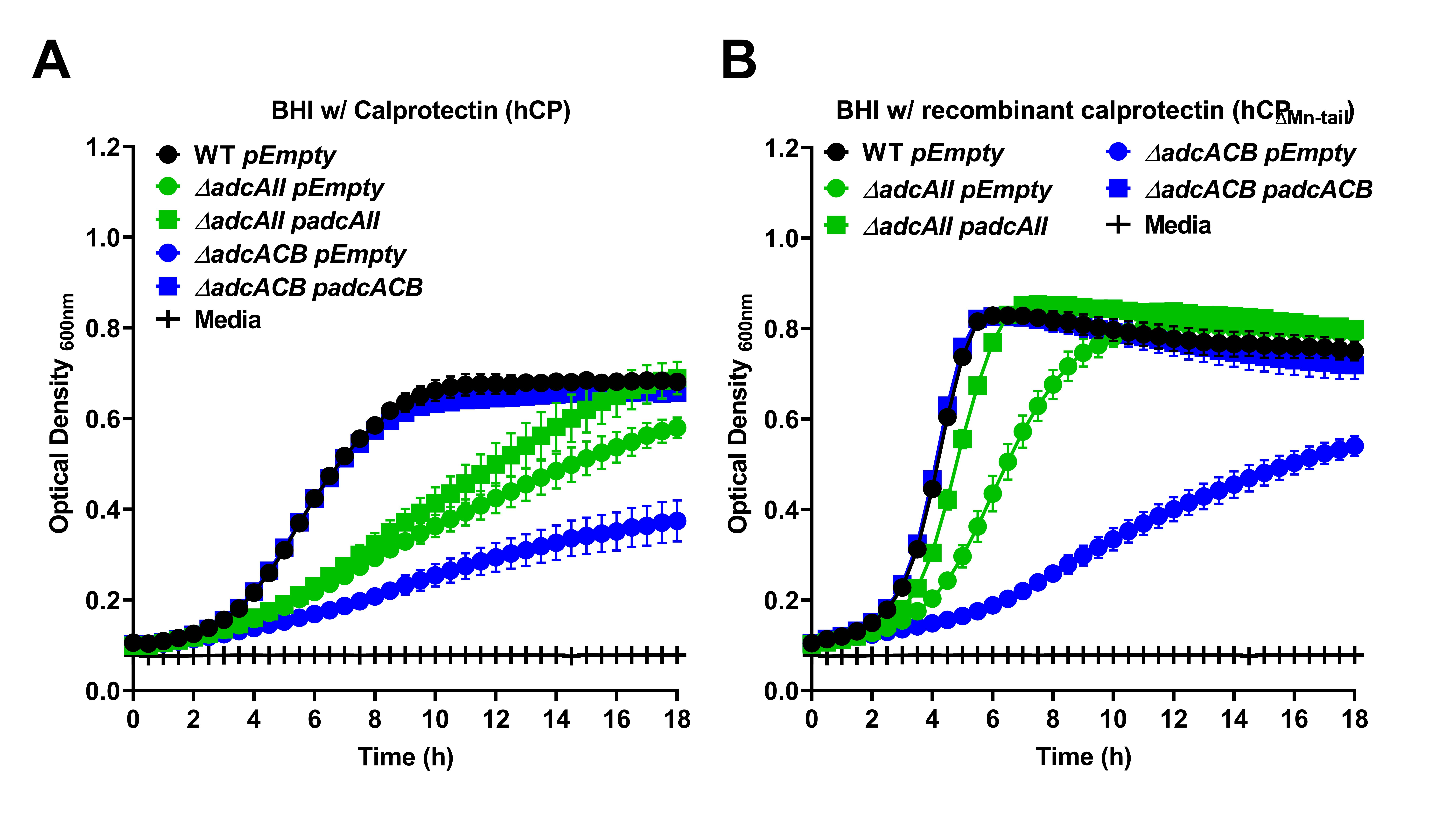

Supplement: Supplemental Material [file KVIR_A_2056965_SM5462.zip › supplementary/Fig S4.jpg]
